# Menstrual patterns and disorders among secondary school adolescents in Egypt. A cross-sectional survey

**DOI:** 10.1186/s12905-015-0228-8

**Published:** 2015-09-04

**Authors:** Hatem I. Abdelmoty, MA Youssef, Shimaa abdallah, Khaled Abdel-Malak, Nawara M. Hashish, Dalia Samir, Moutafa Abdelbar, Ahmed Naguib Hosni, Mohamed Abd-El Ghafar, Yasser Khamis, Mostafa Seleem

**Affiliations:** Department of obstetrics& gynecology, Faculty of Medicine, Cairo University, Cairo, Egypt; Egyptian International Fertility IVF-ET center, 16 Elhassan Ben Ali, Nast City, Cairo, Egypt; Department of Obstetrics& Gynecology, Beni-Suef University, Beni-Suef, Egypt

## Abstract

**Background:**

To our knowledge, no large population – based studies have been performed on the topic of menstrual patterns among Egyptian adolescent in recent years. The aims of this study were to identify menstrual patterns and associated disorders as well as the sources of menstrual health knowledge among Egyptian adolescents.

**Methods:**

A cross-sectional survey. A total of 800 questionnaires were administered to post-menarcheal Egyptian adolescents attending secondary schools in Giza, Egypt, from September 1, 2012, to December 1, 2013. Participants were asked to respond to a semi-structured questionnaire on menstrual health awareness. The questionnaire included items on girl’s socio-demographic and menstrual pattern characteristics, concerning their age at menarche, menstrual cycle length and regularity, duration and amount of flow, type and severity of pain related to menstruation, need for analgesia; and symptoms suggestive of premenstrual syndrome (PMS) Main Outcome Measure: description of menstrual patterns, disorders and source of knowledge.

**Results:**

Four hundred twelve (51.5 %) out of 800 adolescents completed the questionnaire. The mean age of the girls was 14.67 ± 1.7 years. Mean age at menarche was 12.49 ± 1.20 years. 382 respondents reported various menstrual disorders, giving a prevalence rate of 95 %. Dysmenorrhea was the most prevalent (93 %) menstrual disorder in our sample, followed by PMS (65 %), and abnormal cycle lengths (43 %)**.** Menstrual disorders interfered with social and academic life of 33 and 7.7 % of respondents respectively. Most participants lacked menstrual health knowledge and only 8.9 % of girls reported consulting a physician.

**Conclusion:**

To the best of our knowledge, this is one of the largest studies on menstrual pattern and disorders among Egyptian adolescent girls. Our Findings of the present study are consistent with other studies and reported higher than expected prevalence of menstrual disorders.

## Background

Nearly one in four (22 %) Egyptians is an adolescent (ages 10–19) and young adults as a whole (ages 10–24) make up about one-third of the population—just over 20 million persons. Roughly half of these are females [1]. Menstrual disorders among adolescent girls are common due to relative immaturity of the hypothalamic-pituitary-ovarian (HPO) axis, however the exact incidence is unknown since teenagers do not present due to embarrassment as well as lack of knowledge about what is ‘normal’. Even though serious gynecological pathology is rare in this age group ,menstrual problems can be symptoms of certain conditions like polycystic ovarian syndrome and endometriosis, which if undiagnosed and untreated may have profound long-term sequelae in adult life [2]. Few sporadic local studies were conducted to gather data about the frequency of menstrual disorders and their impact on the health status, quality of life, and social integration among Egyptian girls [3–6]. This paucity of data and the secretive nature of menstruation sustain the belief that adolescents’ menstrual complaints are a minor health concern. In addition, the authors' daily observation about misconceptions or incorrect knowledge about menstrual and reproductive health issues; rarity of community based gynecology and pediatrics research about the study subject; and the results of a recently published article by El-Gilany et al. [7] prompted this study. The authors of this study evaluated only the prevalence of dysmenorrhea among adolescent students without considering other menstrual patterns and disorders or exploring source of knowledge among such a group of population. Thus the aim of our study was to identify menstrual patterns and associated disorders as well as the main sources of knowledge about menstrual health matters among a rarely studied segment of the population.

## Materials and methods

This cross-sectional study was carried out in Giza citythe third largest city in Egypt with 6,272,571 population and it is located on the west bank of the Nile)- from September 1, 2012, to December 1, 2013, with female primary and secondary-school students enrolled in public schools. The study was approved by the institutional Ethics Committee of departments of obstetrics and gynecology, faculty of Medicine, Cairo University and was conducted upon approval of the City National Education Directorship and explanation of its objectives to the principals, teachers, and students. Two randomly selected secondary schools agreed to participate in the study. All girls who had already experienced their first menstruation were invited to participate and consecutive consenting post-menarcheal girls aged 11–19 years were surveyed. Informed written consent was obtained from the students and their parents/guardians. A self-administered pretested, questionnaire in Arabic with mostly close ended questions and rating scales was used. Participants were asked to obtain paternal consent and were informed of their right to withdraw from the study at any time. They were instructed not to write their names on the questionnaire and were told that their responses would be confidential.

The aim of the study was explained to the students by one of the researchers. The survey was conducted during school hours at the end of class in trickles following dismissal of male students from September 1, 2012, to December 1, 2013. The 438 Girls who agreed to participate were requested to self-administer a questionnaire inquiring about socio-demographic and menstrual pattern characteristics. Initially girls were asked about their understanding of the function of menstruation and whether they perceived their menstruation to be normal/abnormal. Details of the menstrual history in the questionnaire included; age at menarche, menstrual cycle length and regularity, duration and amount of flow, type and severity of pain related to menstruation; and need for analgesia. They were asked to indicate the severity of pain on a scale from 0 to 10 (0-3 = no/mild pain, 4-7 = moderate pain, and 8-10 = severe pain). Symptoms suggestive of PMS were included in the questionnaire so that the students could check those relevant to them. Menstrual cycle patterns were defined as follows; regular menstrual cycles: cycle length of 21-35days, irregular menstrual cycles: varying cycle length less than 21 or more than 35 days, secondary amenorrhea: having missed ≥3 consecutive cycles in the last 12 months, prolonged menstrual flow (menstrual flow of more than 7 days),and hypomenorrhea (menstrual flow less than 2 days).

Respondents were also asked about the impact of menstruation and its disorders on their school attendance and social life (home chores, sports, going out, social activities). Other variables of interest included; any additional menstrual related symptoms or gynecologic symptoms not mentioned in the questionnaire: sources of information regarding menstrual health knowledge: menstrual hygiene practices and type, dosage, effectiveness and sources of treatment received if required.

Data were expressed as mean ± SD or as a number (percentage) when appropriate. All statistical calculations were done using computer programs SPSS (Statistical Package for the Social Science; SPSS Inc., Chicago, IL, USA) version 15 for Microsoft Windows.

## Results

In all, 800 questionnaires were distributed, 362 girls declined participation and the remaining 438 female students returned the questionnaire; Out of these 412 questionnaires were properly filled, giving a response rate of 51.5 %. Ten girls had pre-existing medical disorders that could affect data interpretation and hence these questionnaires were excluded from further analysis.

The general characteristics of the participants and sources of menstrual health knowledge are shown in Table 1. The mean age of respondents was 14.67 ± 1.7 years with a range of 11–19 years. The median (± SD) BMI for this population was 26.93 ± 3.31 kg/m2. Most of the respondents (92.7 %) were urban residents. College/University education was the highest level of educational attainted by respondents’ mothers (64.2 %). Twenty three respondents (5.7 %) had illiterate mothers.

**Table 1:** General characteristics of respondents and sources of menstrual health knowledge

	Frequency N (%)
Age (years)	
10–14 years	219 (54.4 %)
15–19 years	183(45.5 %)
BMI	
Underweight (<18.5 kg/m^2^)	32 (7.9 %)
Normal (18.5–24.9 kg/m^2^)	56 (13.9 %)
Overweight and above (≥25 kg/m^2^)	314 (78.1 %)
Residence	
Urban	373 (92.7 %)
rural	29 (7.2 %)
Mother’s level of education	
College/University education	258 (64.2 %)
Secondary education	105 (26.1 %)
Primary education	16 (3.9 %)
No education	23 (5.7 %)

BMI, body mass index

Most respondents 370 (92 %) menstruated by the age of 15. The mean onset of menarche was 12.49 ± 1.20 years with a range of , 8 to16 years. Typical menstruation consisted of: regular menses in 66 % (*n* = 265/402) of girls; a cycle length ranging from 21 to 35 days (mean, 27.10 ± 1.58 days) for 57 %; and the mean duration of menstruation was 5.0 ± 1.50 days with a range of 2–8 days. Three hundred and seventeen (78.8 %) of the respondents used sanitary pads, while the remaining 85 (21.1 %) used other items such as tissue paper or towels/cloth. Girls reported changing pads or towels twice 176 (43.7 %), three times 155 (38.5 %), or 4 times 71 (17.6 %) a day respectively.

The pattern of menstrual related morbidities, additional symptoms reported by participants and treatment patterns are shown in Table 2; 382 respondents reported various menstrual disorders, giving a prevalence rate of 95 %. Dysmenorrhea was the most prevalent (93 %) menstrual disorder in our sample, followed by PMS (65 %), and abnormal cycle lengths (43 %)**.** Sixty seven (16.6 %) of the participants reported spotting in between periods and 37 (9.2 %) reported blood clots with menstrual bleeding. Fatigue, mastalgia and mood disturbance before or during a period were the most frequently reported PMS symptoms in our population (68 %, 56 %, 55 %) respectively.

**Table 2:** Menstrual patterns, related morbidities, additional symptoms & treatment patterns in 402 respondents

	Frequency N (%)
Menstrual cycle length (days)	
≤ 20	60 (14.9 %)
21-35	230 (57.2 %)
> 35	112(27.8 %)
Duration of menstrual flow (days)	
< 2	8.0 (1.9 %)
2-6	375 (93.2 %)
7 and above	19 (4.7 %)
Prevalence of menstrual disorders	382(95 %)
Types of menstrual disorders: dysmenorrhea, PMS and abnormal cycle length with the relevant data supplied	
Dysmenorrhea	374(100 %)
Mild	108 (28.8 %)
Moderate	183 (48.9 %)
Severe	83 (22.1 %)
PMS (%)	
Yes	262 (65.1 %)
No	140 (34.8 %)
Abnormal cycle lengths (%)	
Yes	172 (42.7 %)
No	230 (57.2 %)
Missed activities	
Yes	133 (33 %)
School absenteeism (%)	
Yes	31 (7.7 %)
Use of drugs for menstrual disorders	354 (88 %)
Self-medication	249 (62 %)
Treatment given by parents	93 (23.1 %)
Treatment given by a doctor	12 (3.2 %)
Additional symptoms	209 (51.9 %)
Acne	99 (24.6 %)
Hirsutism	69 (17.1 %)
Vaginal discharge	23 (5.7 %)
Vulvar itching	6.0 (1.4 %)
Dysuria	12 (2.9 %)
Information source regarding menstruation (%)	
Mom/sister	312 (77.6 %)
Friends	16 (3.9 %)
Doctor/nurse	3 (0.7 %)
Mass media	15 (3.7 %)
Nobody	56 (13.9 %)

Of the 93 % of girls who reported pain with menstruation, nearly 29 % of the respondents (*n* = 108) reported no/mild pain (0–3on rating scale), 49 % (*n* = 183) reported moderate pain (4–7) and 22 % (*n* = 83) reported severe pain (8–10).

Overall, menstrual disorders prevented 33 % (*n* = 133) of adolescents from participating in social activities and 7.7 % (*n* = 31) from attending school. The mean duration of school absenteeism was 1.5 ± 1.2 days. Dysmenorrhea was responsible for the highest rate of school absenteeism (61 %) followed by PMS (38 %). The majority of absences (58 %) were for 1 day, with 32 % for up to 2 days and 3 girls reported missing up to 4 days of school each cycle..

## Discussion

Three hundred and sixty eight girls declined participation giving a response rate of 51.5 %. This response is considerably low compared to other studies [8–10]. This was mostly due to mothers and girls refusal to participate and is related to cultural menstrual taboos—that menstruation is “dirty,” that it must be hidden and should not be discussed in public. However, this result is not surprising because beliefs about menstruation are usually quite negative in many cultures [11, 12]. Mothers and health educators should encourage girls to feel more comfortable with their bodies teaching them to view menstruation as a maturational event, rather than a hygienic crisis and to reject shameful views regarding a biological female process.

This study sought to provide information on the typical menstrual pattern among Egyptian adolescent school girls. The mean age of menarche was 12.49 years, mean duration of menstrual flow was 5.0 days, and mean frequency of menstruation was 27.10 days. When compared with those from some European and American series and with other data from African countries [13–16], Egyptian girls have one of the earliest recorded means of all populations studied. This could be attributed to the high body mass index of the girls in the sample population [5]. However because of paucity of local data [3, 4], we were unable to comment on trends of age at menarche.

Abnormal cycle lengths occurred in 43 % of our respondents which is higher than the range of 13.2 % to 37.2 % noted by some studies [17–19]. Adolescents have a greater degree of variability in terms of length of inter-menstrual duration due to immaturity of the HPO axis which can take up to 2 years to mature, with increasing frequency and amplitudes of pulsatile GnRH release [2]. The high rate of cycle length abnormalities reported can be explained in part by the mean age of our respondents 14.67 ± 1.7 years. Additional contributory factors include the differences in methodology, and variations in study populations. Nevertheless in cases where abnormal bleeding has occurred greater than 2 years from menarche, other diagnoses must be considered such as PCOS, eating disorders, bleeding disorders, ovarian tumors, and thyroid dysfunction [20].

Dysmenorrhea was a significant problem in our study population with 93 % of respondents reporting various degrees of menstrual pain. This is higher than the 80 % reported by El-Gilany et al. [7], from a sample of 664 Egyptian teenagers and similar to the upper value in the range 59–93 % commonly reported for the same age group [8–21]. Severe pain in our study (22 % of girls) was consistent with the 14–25 % reported in previous studies [8, 22, 23]. The differences in the degree of pain severity may be related to cultural differences in pain perception, absence of a universally accepted method of defining dysmenorrhea and individual variability in pain threshold.

PMS was the second most common menstrual related symptom experienced by our study subjects. Approximately two thirds of all respondents reported one symptom and nearly half reported two symptoms. To our knowledge no previous studies examined the prevalence of PMS among Egyptian adolescents in spite of the high prevalence of PMS symptoms among adolescents reported in this study and similar studies [24, 25].

Approximately one third of our respondents reported additional symptoms (Table 2). Although these were not verified by direct interviewing, examination or investigations, they are important issues to be appropriately managed. Of interest is the high rate of adolescents reporting androgen excess signs (36 %) which in presence of infrequent cycles could be a sign of polycystic ovarian disease. Failure to address these early physical concerns and their reproductive implications can have profound lifelong consequences.

Eighty five percent of the girls perceived their cycle as being normal in spite of the high prevalence of menstrual disorders reported. When asked about source and function of menstruation, approximately 9 % of the respondents answered correctly with the majority stating that it has a “cleansing” effect ridding the body from “dirt”. Statements shaped by the largely negative cultural beliefs. Neither maternal level of education nor residence of the study participants affected the level of knowledge about menstruation. These findings demonstrate that many teens lack the information necessary to recognize their symptoms as medical disorders that can be treated.

None of the girls in our sample reported school education as a source of menstrual health knowledge . This highlights the need to revise the school curriculum as the current educational practices often present girls with primarily biological information lacking more practical information about the many variables that affect menstruation and different copying strategies. This is in agreement with the findings of Koff, Rierdan and others since the 1990 s [26–28], who stated that disconnection between knowledge and a girl’s own body experience occurs when education focuses only on the biological and hygienic aspects of menstruation. It seems that not much has changed since then.

Only 3.7 % of girls reported mass media as source of menstrual health information . This could be related to the media perpetuating certain beliefs, depicting menstruation as a hygienic crisis and as a secret event resulting in a culture of concealment surrounding menstruation. This may explain, at least in part, the low response rate in the current study. Another significant finding of this study is that only 0.7 % of adolescents reported receiving information regarding menstruation from a health care professional. As a result adolescent girls in our study are missing the opportunity to receive health education about menstrual problems, as well as their diagnosis and treatment. Development of health assessment forms with standardized menstrual history questions may serve as a reminder for health care providers to discuss menstruation with adolescents.

Interference of menstrual symptoms with school attendance and social life observed among our respondents has been reported by others [8, 17]. Activities most commonly limited were home chores, participation in sports and social events, relationship with family and friends and homework tasks . Interestingly school attendance was the least activity affected by menstrual problems reported only by 7.7 % respondents. This is comparable to the findings of El-Gilany et al. [7]. All the limitations were significantly more frequent among students with dysmenorrhea.

Adolescents in the present study used a variety of measures to cope with menstrual cycle problems; pharmacologic (46 %), non-pharmacologic (24.4 %) and consulting a physician (8.9 %). Medications were self-administered, provided by respondents’ parents and health care professional in (62, 23, and 3 %) of cases respectively. Similar to other studies [29, 30], non-steroidal anti-inflammatory drugs (NSAIDs), paracetamol and aspirin were the most frequently used pain medications. Other measures used by respondents to alleviate menstrual pain included; use of a heating pad (40.5 %), rest (21.0 %), uncategorized herbal drinks (17.0), exercise (12.6) and 2 % used non-contraceptive hormonal medications. Of those respondents who took analgesics, 75 % reported moderate to high effectiveness (scoring 4–10 on a scale of 0–10, where 0 = not effective and 10 = highly effective). Only 2.9 % of all respondents reported having been treated for menstrual cycle irregularities. This was mostly done with unspecified medications.

Alarmingly while effective treatments for menstrual disorders exist; approximately two thirds of all respondents self-medicated, one quarter reported low effectiveness of analgesics used and only 7 % consulted their health care provider. This latter figure is comparable to other studies [23, 30] and is of significant concern because if these disorders are considered to be “normal” and ignored, teenagers may not seek care for underlying gynecologic conditions. Additional studies are needed to increase our understanding of coping strategies used by girls with menstrual problems and their reluctance to access medical treatment.

Our study has some limitations that need to be addressed. The present study comprised adolescents with certain demographic criteria (e.g. Formal education, mainly urban residence), which could be seen as a limitation. Further research with adolescents who have different socio-cultural characteristics is needed to generalize the results. Although, we did not calculate sample size because it is the first study to be conducted on Egyptian adolescent’s girls, the large size of our sample probably sufficed to enable a robust analysis of the menstrual outcomes. Additionally the data could be affected by recall bias which is common to all questionnaire-based studies. However, people tend to recall experiences that are most relevant to them.

## Conclusion

To the best of our knowledge, this is one of the largest studies on menstrual pattern and disorders among Egyptian adolescent girls. Our Findings of the present study are consistent with other studies and reported higher than expected prevalence of menstrual disorders. It highlights the need for culturally sensitive menstrual health education programs and school based screening services targeting this important segment of population with active involvement of reproductive health specialists, gynecologists, pediatricians, teachers and school nurses. It also identifies the need for utilization of the influential role of mass media for awareness creation and facilitation of the dissemination of menstrual health information especially among uneducated adolescents.

## Abbreviations

HPO: Hypothalamic pituitary ovarian axis
PMS: Premenstrual syndrome
PCOS: Polycystic ovary syndrome
